# Barriers to blinding: an analysis of the feasibility of blinding in test-treatment RCTS

**DOI:** 10.1186/1745-6215-16-S2-O30

**Published:** 2015-11-16

**Authors:** Lavinia Ferrante di Ruffano, Jon Deeks

**Affiliations:** 1 University of Birmingham, Birmingham, UK

## 

Test-treatment strategies are complex interventions involving four main ingredients: 1) testing, 2) diagnostic decision-making, 3) therapeutic decision-making, 4) subsequent treatment. Methodologists have argued that it may be impossible to control for performance bias when evaluating these strategies using RCTs, since test results must be used by clinicians to plan patient management, whilst patients are often actively involved in testing processes and treatment selection. Analysis of complex therapeutic interventions has shown blinding is not always feasible, however claims regarding the ability to blind in test-treatment trials have not been evaluated.

This methodological review analysed a systematically-derived cohort of 103 test-treatment trials to determine the frequency of blinding, and feasibility of blinding care-providers, patients and outcome assessors. Judgments of feasibility were based on subjective assessments following previously published methods.

Care-providers, patients and outcome assessors were masked by 4%, 5% and 22% of trials, and could have been masked by a total of 11%, 50% and 66% respectively. Scarcity of attempts to blind reflected the practical and ethical difficulties in performing sham diagnostic procedures, or masking real test results from patients and clinicians. Feasibility hinged on: the types of tests, nature of their comparison, type of information produced and circumstances surrounding their administration.

These findings present worrying implications for the validity of test-treatment RCTs. Unexpectedly we found that in some circumstances blinding may alter or eliminate the desired test-treat effect, and recommend further investigation to determine the true impact of masking in these highly complicated trials.

